# Comparative proteomics reveal characteristics of life-history transitions in a social insect

**DOI:** 10.1186/1477-5956-5-10

**Published:** 2007-07-17

**Authors:** Florian Wolschin, Gro V Amdam

**Affiliations:** 1 Arizona State University, School of Life Sciences, PO Box 874501, Tempe, Arizona 85287, USA; 2 Norwegian University of Life Sciences, Dept. of Animal and Aquacultural Sciences, PO Box 5003, Aas N-1432, Norway

## Abstract

**Background:**

Honey bee (*Apis mellifera*) workers are characterized by complex social behavior. Their life-history is dominated by a period of within-nest activity followed by a phase of long-distance flights and foraging. General insights into insect metabolism imply that foraging onset is associated with fundamental metabolic changes, and theory on social evolution suggests metabolic adaptations that are advantageous for the colony as a whole.

**Results:**

Here we address the life-history characteristics of workers with LC-MS/MS based relative quantification of major proteins. Our approach includes: i. Calculation of a false positive rate for the identifications, ii. Support of relative protein quantification results obtained from spectral count by non-parametric statistics, and iii. Correction for Type 1 error inflation using a bootstrap iteration analysis. Our data are consistent with the use of glucose as the main fuel for honey bee flight. Moreover, the data delivers information on the expression of ATPsynthases/ATPases, and provide new insights into nurse- and forager-specific patterns of protection against oxidative stress.

**Conclusion:**

The results show the suitability of this approach to investigate fundamental biochemical changes in an insect, and provide new evidence for metabolic specializations that occur during the social ontogeny of worker honey bees.

## Background

Studies of the biochemistry of life-history progression provide insights into the dynamic properties of biological systems, including metabolic change [1]. A fundamental step in evolution was the emergence of social life, as exemplified by social insects [2]. The best-studied insect model in sociobiology is the honey bee, which has proven useful for understanding regulatory changes that may have occurred during social evolution [3-7]. Work on honey bee life-history progression has focused on the transition from nest-tasks to flight activity and foraging, a behavioral shift characteristic of the caste of facultative sterile worker females (reviewed by [8]). Several studies have demonstrated changes in brain mRNA levels when worker bees shift from working in the nest to foraging flights approximately 2–3 weeks after adult emergence [9-12]. Differences are found for genes involved in signal transduction and primary metabolism [13], but mRNA expression does not always correlate with protein concentrations and metabolic state [14-17].

Enzyme activity measurements and flux analyses on worker bees, including studies of hexokinase, phosphofructokinase, glyceraldehyde-3-phosphate-dehydrogenase, citrate synthase and cytochrome oxidase, indicate that oxidation of carbohydrates is the primary energy source used for foraging flights (e.g. [18-20]. Also, recent use of a 2d-gel based proteomic approach in combination with enzyme assays point to metabolic changes in honey bee thorax muscle that can increase flight ability after foraging onset (increase in troponin T 10a and citrate synthase, [21]). We address this important life-history transition by using an electrospray tandem mass spectrometry approach for relative quantification of the whole-body proteome of nest workers and foragers. A common argument against whole-body analyses is that the contributions of different organs or tissue types to the overall pattern are unclear. Yet whole-body analyses have provided fundamental insights into key biological processes like aging, development, and immunity (e.g. [22-24]: They focus quantitatively on the most important changes in the organism as a whole, and complement more targeted tissue- or organ-specific profiling methods.

Although not routine, recent descriptions of some honey bee subproteomes (subset of the whole proteome of an organism, i.e. royal jelly proteome, bee venome proteome, hypopharyngeal gland proteome, haemolymph proteome), prove that proteomic research on honey bees using mass spectrometry is feasible [25-28]. Our approach included an estimate of the false positive rate, non-parametric statistics, and iterative correction against Type 1 error inflation. In total, 113 proteins were identified and 47 of them quantified by our approach. Levels of 15 proteins diverged significantly between nest workers and foragers. Our data are consistent with the use of glucose as the main fuel for honey bee flight. Moreover, the data delivers information on the expression of ATPsynthases/ATPases, and provide new insights into nurse- and forager-specific patters of protection against oxidative stress.

## Results and discussion

### Methodology setup and validation

Experimental manipulation and data analysis at a systemic, whole body level can readily increase our knowledge about living organisms. This principle is apparent e.g. when looking at the outcome of systemic RNA interference approaches (e.g. [29,30]). Here, tissue specific effects are not well defined but global response can be monitored and understood. If differences can be observed on the whole-organism level they are likely to be substantial, as they are robust to deflation by combining body compartments. Further, although separating an organism into its parts can help to clarify how contributions of different tissues translate into global patterns, the data cannot easily be added up to the overall pattern of any protein. This is because background matrix will vary from tissue to tissue and might impair quantification itself. Therefore, whole body analyses can contribute in a unique way to increase our knowledge about biochemical changes during honey bee ontogeny.

Our approach consists of a protein extraction step, followed by protein digestion, separation of peptides via HPLC and characterization of the proteins by mass spectrometry. We were able to identify 113 proteins and several hundred peptides with this approach [see Additional files 1 and 2]. Relative quantification is accomplished by spectrum count where the spectra that are identified for any given protein in one sample are counted and this value is compared to the value obtained for the same protein in another sample [31-35]. Thus, every quantified protein was identified several times leading to a high reliability of identification.

**Figure 1:**
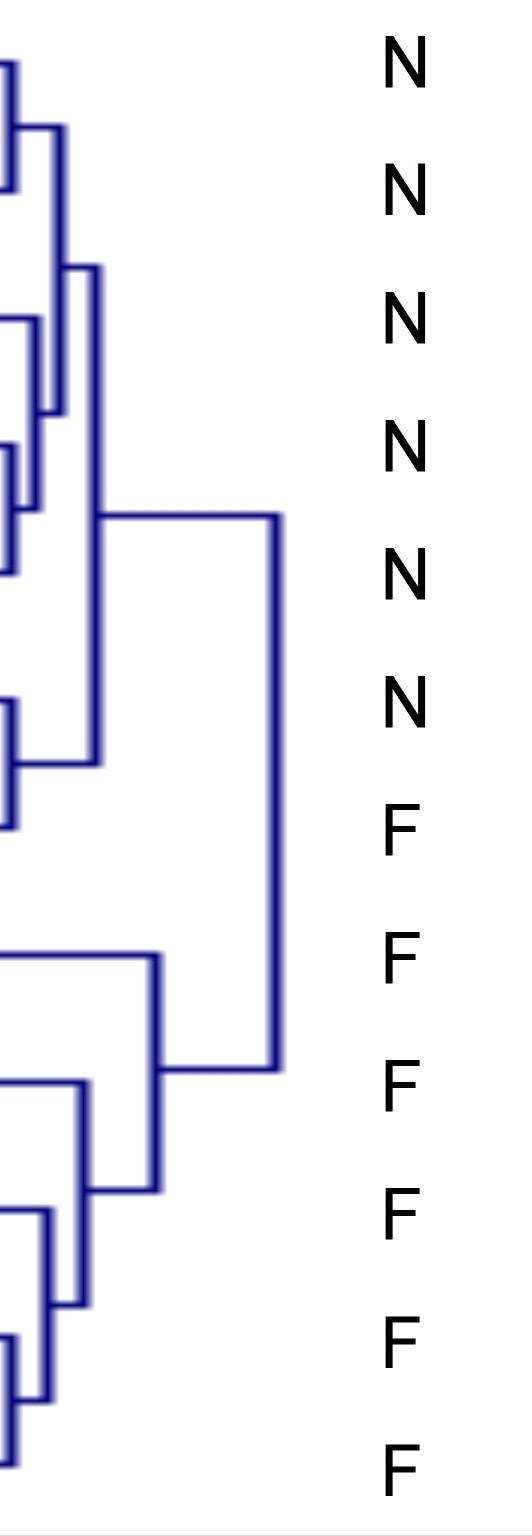
Result of a hierarchical clustering analysis computed on quantifiable proteins.

In our experiment, actin showed the most stable protein quantity over all samples and was used for standardization. Use of honey bee actin for relative quantification is also common in mRNA expression studies (e.g., [3]). We further validated our approach to data handling by standardization toward the overall spectral count obtained for each sample. Statistics were in good agreement between these two methods of correction [see Additional file 1], and even without standardization more than 75% of the observed differences in protein expression remained (data not shown). Our analysis, therefore, is both reproducible and robust, and in the following we report the results based on correction toward actin. Of the 47 quantifiable proteins, 15 were expressed at significantly different levels in nest bees and foragers [as determined by Mann-Whitney U-test, *P *< 0.05, *n *= 12, see Additional file 1]. A hierarchical clustering analysis computed on the quantifiable proteins revealed distinct clustering of behavioral groups with one exception [Figure 1]. Surprisingly, one forager clusters much closer to the nest bees than to the other forager bees. This could be because the respective bee was on the verge of changing its tasks (bees in this study were of unknown age) what would imply that some physiological features of the nest bee stage are retained in young foragers and that there is a gradual rather than an abrupt change in the expression pattern of some proteins when bees change from in-hive tasks to foraging.

### Glucose consumption

The main task of foragers is to provide the colony with nectar and pollen. Consequently, one of their major behavioral characteristics is long-distance flights for the purpose of food collection. There is current agreement that honey bee flight is predominately fueled through hexose sugars [20,36]. Thereby, primarily glucose is used to produce ATP and NADH necessary for the exhaustive demands of the flight process. The bee thorax is largely dominated by flight muscle tissue [37], and proteins of the thorax make up a major fraction of total bee protein [38]. In addition, metabolic rate during flight is much higher than during non-flight activities [39], and activities of glycolytic enzymes increase from the nest bee stage to the forager stage [40]. These observations led us to expect that in honey bees the concentrations of proteins in the major pathways of ATP production and use can reflect flight muscle contents, and that they should be higher in foragers than in nest bees. This higher level would indicate a major physiological adaptation associated with the shift in social behavior from nest tasks to intense flight activity.

**Figure 2:**
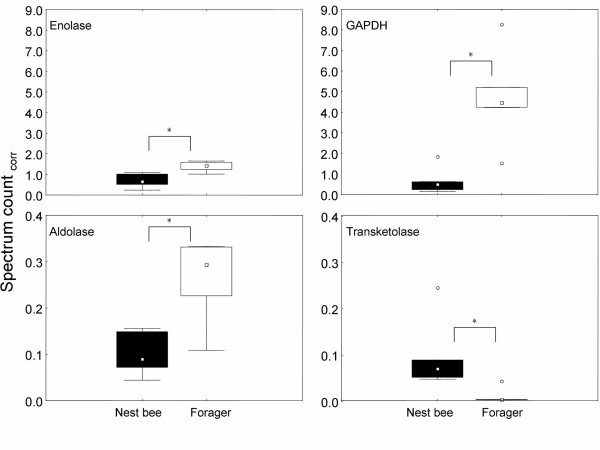
Box-and-whisker-plots of selected proteins associated with glucose processing Boxes represent 25–75% percentiles of the data, outliers are marked as open circles. * denotes significant differences between nest bees and foragers, for p-values see Additional file 1. Aldolase: fructose-1-6-bisphosphate aldolase, GAPDH: glyceraldehyde-3-phosphate-dehydrogenase. Spectrum count_corr_: spectrum count corrected for actin.

In accordance with this prediction, we found that proteins similar to the glycolytic enzymes fructose-1,6-bisphosphate aldolase, glyceraldehyde-3-phosphate-dehydrogenase (GAPDH) and enolase are significantly elevated in foragers [Fig. 2].

### Food synthesis and processing

Nest bees and foragers have different provisioning functions. Nest bees care for the brood and queen, whereas foragers bring in the resources needed for colony growth and reproduction. Unlike foragers, nest bees synthesize proteinaceous jelly in a set of paired acinous head glands (hypopharyngeal glands), and these secretions are fed to the brood and to other adult bees including foragers [41]. A family of proteins specific to jelly are the major royal jelly proteins [42,43]. In general, the bee's biology of jelly synthesis and transfer matches well with our finding of higher values for a major royal jelly protein in nest bees compared to foragers [see Additional file 1]. We also observed that a protein similar to a transketolase, which is an important enzyme in the pentosephosphate-pathway, is downregulated in foragers [Fig. 2]. One of the major functions of this pathway is the production of NADPH and building blocks that can be used in biosynthetic reactions [44]. This may point to a more important role of reactions involving NADPH in nest bees compared to foragers.

To fuel flight, foragers must utilize high energy containing food. Their capacity of glycolysis appears to be upregulated compared to nest bees (described above). However, we reasoned that enzymes involved in sugar uptake and processing would also be upregulated. Interestingly, a protein highly similar to an alpha-glucosidase, a protein that is involved in the processing of disaccharides to glucose, shows higher levels in foragers [see Additional file 1]. This result agrees well with previous reports by Kubo et al. on elevated levels of alpha-glucosidase mRNA and protein in foragers [45-47]. We conclude that our observations generally agree with a higher capacity of sugar processing and NADH production in foraging honey bees.

### Citric acid cycle enzymes

Pyruvate produced by glycolysis is converted to acetyl-CoA and enters the citric acid cycle, which serves further production of reduction equivalents and biosynthetic precursors. On an organismal level, our data suggest that the dynamics of the honey bee citric acid cycle enzymes are complex. While a malate dehydrogenase-like protein is upregulated in foragers, the amounts of other enzymes of the cycle including an isocitrate dehydrogenase-like protein and a protein similar to oxoglutarate dehydrogenase remained seemingly unchanged [see Additional file 1]. These results suggest that some enzymes of the citric acid cycle can be regulated by metabolites or posttranslational modifications rather than on the level of enzyme amount [48], that higher expression levels in one tissue are accompanied by lower expression levels in another tissue, that differences were too subtle to be detected by the method we used, and/or that malate dehydrogenase has an additional role besides its involvement in the citric acid cycle, e.g. its function in an aspartate/malate shuttle [49]. Interestingly, Sacktor *et al*. reported a sharp increase of malate concentration at the beginning of flight in blowfly while levels of citrate, oxaloacetate and α-ketoglutarate remained seemingly unchanged [50]. However, no explanatory framework was provided with their observation.

One major consumer of NADH generated by glycolysis and the citric acid cycle is the mitochondrial respiratory chain. While electrons are transported from NADH to oxygen, an electrochemical gradient is build up over the inner mitochondrial membrane. This gradient in turn is used to drive the generation of ATP by ATPsynthases. These proteins, accordingly, play a key role in production of ATP. As muscle activity depends largely on the availability of ATP, and as an increase in mitochondria and cytochromes from young nest bees to older foragers was reported by Herold and coworkers [51,52], we expected higher levels of respiratory chain enzymes in foragers. The lack of differences in cytochrome c-levels (a protein of the mitochondrial respiratory chain) was thus surprising. Notably, the overall spectrum count for cytochrome c was low, and therefore the lack of differences between nest bees and foragers could to be due to methodological limitations. A change in the protein extraction technique could enable improved insight into these dynamics in future analyses.

### Enzymes related to ATP generation and consumption

**Figure 3:**
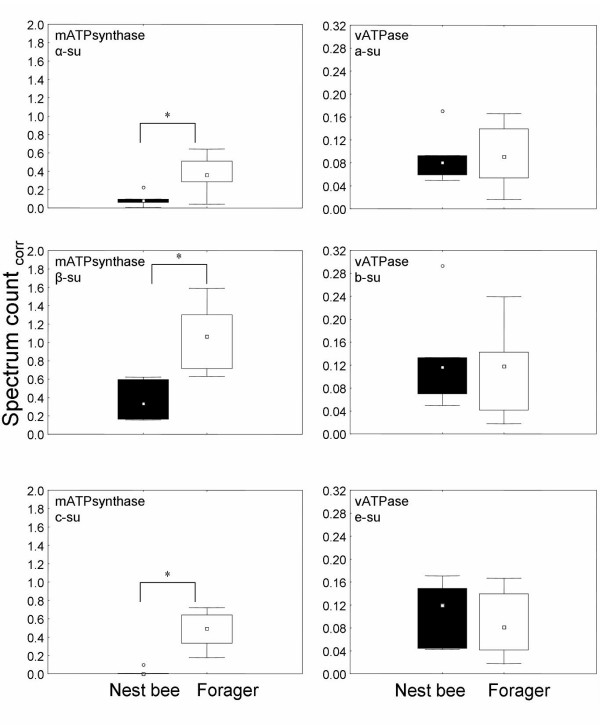
Box-and-whisker-plots of proteins homologues to ATPases/synthases. Boxes represent 25–75% percentiles of the data, outliers are marked as open circles. * denotes significant differences between nest bees and foragers, for p-values see Additional file 1. Left: subunits (su) of the mitochondrial ATPsynthase. Right: subunits (su) of a v-type ATPase. Spectrum count_corr_: spectrum count corrected for actin.

ATPsynthase subunit homologs were more abundant in foragers, in accordance with foragers having larger ATP production capacities than nest bees [Fig. 3].

In contrast, levels of proteins similar to the subunits of a v-type ATPase remained unchanged [Fig. 3]. V-type ATPases are structurally similar to mitochondrial ATP synthases but serve other functions [53]. Instead of generating ATP, they establish and maintain an electrochemical gradient over plasma membranes under the consumption of ATP [54]. Our data indicate that v-type ATPases are equally important for both nest and forager bees and may illustrate how two structurally similar proteins (mATPsynthase and vATPase) can differ profoundly in their roles in life-history transitions.

The data discussed so far suggest that in foragers a whole pathway involved in the processing of glucose and the generation of ATP and NADH is optimized to meet the demands of the energy consuming flight process. However, we also observed differences that can not be explained by this line of argument. Arginine kinase, for example, converts arginine plus ATP into phosphoargine plus ADP and vice versa [55], and can be used to control the ATP resources and rapidly release ATP when necessary. Indeed, a highly related protein (creatine kinase) is one of the major players in muscle contraction and maintenance of ATP/ADP balance in mammals [56,57]. Arginine kinase activity has been reported in insect flight muscle and its role in maintaining high levels of ATP at the sites of muscle contraction has been discussed [58-60]. Thus, higher arginine kinase activities are often associated with greater locomotory performance. Based on these findings, one could assume that foragers – in constant demand for rapidly released energy, for maintenance of adequate ATP/ADP ratios during flight, and with greater locomotory performance – have higher levels of arginine kinase. Yet we found that arginine kinase levels are significantly higher in nest bees [see Additional file 1]. A possible explanation is that nest workers, which perform defecation flights and also use flight muscle contraction to maintain optimal nest temperature (fanning, warming), utilize more energy phosphates than foragers as their oxidative catabolic pathways may not yet be optimized for flight. However, arginine kinase mRNA is highly expressed in honey bee tissues other than the thorax (head, abdomen) [61], and thus the contribution of flight muscle arginine kinase to whole-body patterns are likely confounded by other sources. Ubiquitous distribution of arginine kinase might suggest its involvement in a synthetic redox/shuttle mechanism that is common to many tissue types.

### Reactive oxygen defense

**Figure 4:**
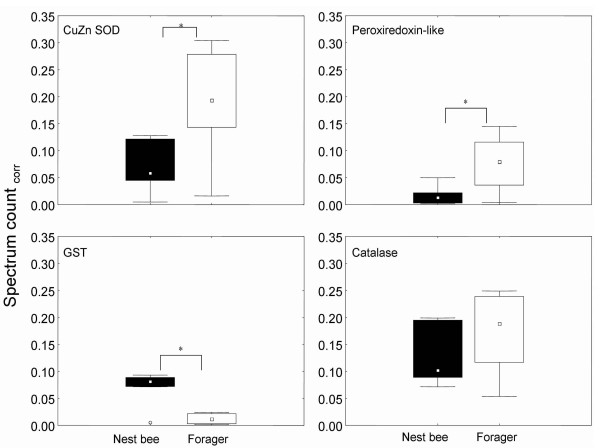
Box-and-whisker-plots of proteins homologues to proteins putatively involved in oxygen stress response. Boxes represent 25–75% percentiles of the data, outliers are marked as open circles. * denotes significant differences between nest bees and foragers, for p-values see Additional file 1. CuZn SOD: CuZn superoxide dismutase. GST: glutathione-s-transferase. Spectrum count_corr_: spectrum count corrected for actin.

One class of proteins that in honey bees appears to deviate from general biochemical reasoning [7,62] is involved in protection against reactive oxygen species (ROS). A major threat for organisms that depend on oxygen for metabolism is production of ROS that cause oxidative damage to cellular components. Due to a high demand for oxygen during flight and, thereby, an assumed increase in ROS and a documented increase in oxidative damage [63]; one might reason that foragers generally invest in higher levels of proteins involved in ROS protection. Interestingly, at the whole-organism level we found no general bias of the relative abundance of proteins putatively involved in the protection against oxidative stress in foragers compared to nest bees [Fig. 4].

Levels of CuZn superoxide dismutase and of a peroxiredoxin-like protein were observed to be at least 3-fold higher in foragers. Homologs of these proteins have been shown to protect mammalian muscle from oxidative damage and this might also be true in honey bees [64]. Other enzymes commonly associated with ROS protection are catalase and thioredoxin reductase, but their levels remained unchanged in foragers [Fig. 4 and Additional file 1]. In sharp contrast, glutathione-s-transferase (GST) levels, another enzyme involved in the response against oxidative stress and in detoxification but also in synthetic pathways [65], were at least 7-fold higher in nest bees. For some insects age-related decrease of GST activities and levels have been reported previously [66-68]. Studies on GSTs in honey bees revealed a complex influence of several parameters on GST activity including colony size, brood/adult ratio [69], insecticide treatment, temperature, and starvation [70]. Smirle and Winston reported that the total detoxification capacity related to GST and mixed function oxidases was lower in forager midguts compared to nest bee midguts [71]. However, since the enzyme activity per milligram midgut protein was higher in foragers, they stated that foragers account for loss of GST levels to some extent by increasing GST activity.

Proteins involved in the defense against ROS have major effects on longevity in many if not all organisms [72,73]. It has been suggested that lack of sufficient ROS defense mechanisms in foragers reflects reduced somatic maintenance, that this reduced maintenance conserves energetic resources at the colony level, and that it in part is responsible for rapid somatic decline in foraging bees [63]. In a recent study, our group showed that nest bees in comparison with foragers are more tolerant to the ROS inducing agent paraquat [7]. This effect was directly and positively linked to the circulating vitellogenin protein levels in the respective bees, and vitellogenin was shown to exhibit characteristic antioxidant function by being preferentially carbonylated by ROS. In the present study we were unable to quantify vitellogenin, although a possible peptide from this protein was detected [see Additional file 2]. This inability to quantify vitellogenin is likely due to relatively low whole-body abundance in the sampled bees. However, our proteomic data suggest that other compounds like GST might contribute to the higher ROS tolerance of nest bees, and that protection mechanisms in foragers may be limited to proteins indispensable for their foraging performance, as we have suggested previously [74-76].

## Conclusion

In this study we compare the overall protein profiles of honey bee nest workers and foragers. The resulting data provide new biochemical insights into the major adult life-history transition of this social insect, although they do not permit the separation of behavioral and age effects. Our findings should encourage future studies on tissue-specific profiles that ultimately can reveal how different parts of the body contribute to the overall proteomic profile, and also show how each tissue contributes to major biochemical processes.

## Methods

### Chemicals

Unless indicated otherwise, chemicals were obtained from Sigma-Aldrich (St. Louis, USA) and Roche (Indianapolis, USA).

### Bee sampling and protein extraction

Nest workers and foragers were collected from 2 wild type (unselected) honey bee colonies in the apiary of Arizona State University. Bees were sampled based on established behavioral assays: nest workers were collected in the brood nest while inserting their head into cells containing larvae (performing nursing behavior) [77], and foragers were collected at the hive entrances when returning from foraging flights [78]. Nectar and/or pollen loads were determined to confirm forager identity. Bees where then frozen in liquid nitrogen, and stored at -80°C until further use. Protein extraction was essentially performed as described in [79]. Briefly, a protein extraction mixture containing 50 mM hepes pH 7.5, 6 M urea, 40% sucrose, 1% β-mercaptoethanol, and 60 mM sodium fluoride (300 μl/bee) as well as tris buffered phenol (900 μl/bee) was added and bees were ground in this mixture at room temperature followed by incubation at 4°C for 15 min on a sample rotator. Subsequently, samples were centrifuged at 12.000 rpm for 5 min, and 300 μl of the upper phase were precipitated with ice-cold acetone at -20°C over night. On the following day the samples were centrifuged for 5 min at 10.000 rpm and the pellet was washed twice with 300 μl ice-cold methanol each. Finally, pellets were dried at RT for 20 min.

### Protein digestion and sample preparation for HPLC

For tryptic digestion protein pellets were first redissolved in 50 μl of a dissolvation buffer containing 50 mM ammoniumbicarbonate, 8 M urea, and 1 mM CaCl_2_. Then, 150 μl of digestion buffer (50 mM ammoniumbicarbonate, 1 mM CaCl_2_) and 2 μl of a 0.5 μg/μl trypsin solution (trypsin in 1 mM HCl) were added and proteins were incubated over night at 37°C. Samples were desalted on the next day using C18 extraction plates (3 M empore, St. Paul, USA) following the instructions provided in the manual. Desalted peptides were dried in a speed vacuum device and stored at -20°C until further use.

### HPLC and mass spectrometry

Dried peptides were redissolved in 8 μl of 5% acetonitrile, 2% TFA of which 6 μl were used for analyses. Peptides were separated on a monolithic column (100 μm ID, Merck, Darmstadt, Germany) using a 95 min gradient ranging from 95% A (0.1% formic acid, 99.9% H_2_O) to 80% B (0.1% formic acid, 99.9% acetonitrile) followed by a 15 min equilibration step. Peptides were eluted from the reversed phase μLC column directly into an LTQ mass spectrometer (Thermo, San Diego, USA). The isolation window was set to 3 m/z, collision energy to 35, and the activation time to 30 ms. MS^2 ^was triggered for the three most abundant peaks in each MS spectrum. Using the open source search tool OMSSA [80] the spectra were matched against an *A. mellifera *sequence database retrieved from the National Center for Biotechnology Information (NCBI, Bethesda, MD, USA) containing additional trypsin and keratin sequences. The following criteria were used: 0.8 Da fragment tolerance, 2.0 Da precursor tolerance [81], maximum of 2 missed cleavages, only tryptic sequences allowed, only one hit per peptide reported (only the best hit for a given spectrum with a given scan number was shown), variable modifications: methionine oxidation.

To assess the rate of false positive identifications and to obtain an adequate e-value cutoff, peptides were also searched against a database containing the same sequences as the original database but in reversed order. False positive spectra were determined to make up less than 1% at an e-value of 1 (evalue -he 1), which was used in all analyses. In addition, a protein hit was only accepted if at least three spectra were obtained representing two distinct peptides or one single peptide if it represented more than 20% of the protein sequence and displayed an e-value of at least 0.1. Using these additional criteria no false positive protein hits were identified in a reverse database search. When hits with ambiguous identifications were obtained due to the presently unfinished annotation of the *A. mellifera *genome, affiliation of the identified peptides to certain proteins was ascertained using BLAST [82]. Proteins were quantified using spectrum count, which delivers an estimate for relative quantification [31,32,35]. For standardization purposes it was determined which of the proteins displayed the lowest variation throughout all samples. The individual values for this protein were then used to correct protein values by dividing each protein value by the "standard protein" value for the respective sample. In addition, we calculated ratios of individual spectral count of every quantified protein divided by the overall spectral count for every sample. This leads to normalized values that are corrected for overall protein amount. Proteins were only quantified, if accepted (criteria for acceptance as described) in at least four of the six measured samples belonging to one sample group (hive bee or forager). In the remaining samples where no peptides were found for the respective proteins, an arbitrary value of 0.1 was used for quantification calculations.

### Statistics

Of 113 proteins identified, 47 met the criteria for quantification (n = 12, 6 nest bees and 6 foragers, one run each). Of these 47, data for 4 proteins did not conform to assumptions of ANOVA, as determined by Hartley-Cochran-Bartlett and the Levene's test. Therefore, the non-parametric Mann-Whitney U-test was used to test for significant differences between nest bees and foragers. To control the Type 1 error rate, 1000 bootstrap iterations were run for each protein using values corrected for actin amounts (see Results and Discussion). During one iteration, 6 expression values were randomly assigned to each of two groups, and a *P*-value calculated using the Mann-Whitney U-test. The 1000 *P*-values were subsequently sorted in ascending order, and the bootstrap cut-off value of the 5% lower tail was determined for the *P*-value distribution. This cut-off was > 0.13 for all the examined proteins, which implies that our report of significance at an alpha level of 0.05 is not associated with inflation of Type 1 error. The analysis was conducted with Statistica 6.0. The bootstrap algorithm was written in MatLab 6.5.

The hierarchical clustering analysis was conducted on the actin normalized values of the quantifiable proteins. Euclidean distance and average linkage clustering was used to visualize sample groups with common features (TIGR Multiexperiment Viewer version 4.0 b [83]).

## Competing interests

The author(s) declare that they have no competing interests.

## Authors' contributions

FW designed the study and carried out the experimental work. GVA supervised the collection of bees for analysis, guided the non-parametric statistical analysis, and performed the bootstrap correction. FW and GVA wrote the manuscript. All authors read and approved the final manuscript.

## Supplementary Material

Additional file 1 Proteins quantified by spectrum count. Table displaying the results from a non-parametric Mann-Whitney-U-test (rank sums and p-level) for proteins quantified by spectrum count (threshold level for significant differences p < 0.05). Click here for file

Additional file 2 Peptides identified by LC-MS/MS. Table displaying all peptides identified by OMSSA with an e-value ≤ 1.0E-01. Lower case letter m: oxidized methionine. For details on OMSSA see [80]. Click here for file
